# A Case of Petrous Apex Cholesteatoma Removed Using an Exoscope and an Endoscope Without Posterior Rerouting of the Facial Nerve

**DOI:** 10.7759/cureus.104699

**Published:** 2026-03-05

**Authors:** Shinya Ohira, Manabu Komori

**Affiliations:** 1 Department of Otolaryngology, St. Marianna University School of Medicine, Kawasaki, JPN

**Keywords:** cholesteatoma, endoscope, exoscope, otologic surgical procedures, preservation of neural function, subtotal petrosectomy, trans-superior semicircular canal approach

## Abstract

Petrous apex cholesteatoma (PAC) is a rare disease extending into the deep area of the temporal bone. Traditionally, lesions surrounding the geniculate ganglion often required facial nerve rerouting, which carries the inherent risk of worsening facial nerve function due to neural ischemia. We report a case of PAC successfully removed via subtotal petrosectomy combined with a trans-superior semicircular canal approach, achieving complete resection while preserving neural function. A 43-year-old female with a history of surgery for cholesteatoma and pre-existing left-sided deafness presented with left moderate facial nerve palsy and vertigo. CT revealed a PAC extending from the epitympanum to the middle cranial fossa and the superior internal auditory canal. We performed a subtotal petrosectomy combined with a trans-superior semicircular canal approach. The surgery was conducted under exoscopic visualization for safe bone drilling; however, for the deepest lesions located medial to the geniculate ganglion and the labyrinthine segment, which typically require posterior facial nerve rerouting, a 30-degree angled endoscope was utilized. This hybrid approach allowed for complete resection without the need for rerouting. Postoperatively, there was no worsening of facial palsy or vestibular dysfunction. Six months later, there was no evidence of recurrence. For PAC extending deep into the temporal bone, the strategic combination of an exoscope and an angled endoscope enables safe, minimally invasive total resection while avoiding facial nerve rerouting. This approach represents a valuable surgical option that allows complete resection and neural preservation.

## Introduction

Petrous apex cholesteatoma (PAC) is a relatively rare condition (0.1-0.2% among temporal bone cholesteatomas) [1,2]. The petrous bone is located deep within the skull base and is closely adjacent to vital structures, such as the inner ear, cranial nerves, carotid artery, and skull base, among temporal bone cholesteatomas. Due to its extension into the deep structures of the petrous bone, it often poses significant diagnostic and therapeutic challenges. Clinically, PAC frequently remains asymptomatic for extended periods and is typically detected only upon the onset of facial nerve palsy or inner ear symptoms [1]. The management of PAC requires various approaches to the deep temporal bone. In cases where the lesion involves the area surrounding the geniculate ganglion, facial nerve rerouting is often required to achieve adequate exposure. However, rerouting carries an inherent risk of worsening facial nerve function due to compromised neural blood supply; therefore, it is desirable to avoid this procedure whenever possible [3]. In recent years, advancements in endoscopic surgery have enabled increasingly minimally invasive approaches in temporal bone surgery [4]. We report a case of PAC extending deep to the geniculate ganglion, which was successfully managed via subtotal petrosectomy combined with a trans-superior semicircular canal approach. By strategically utilizing both an exoscope and an endoscope [5], we achieved complete resection without facial nerve rerouting, thereby ensuring functional preservation.

## Case presentation

A 43-year-old female presented with a primary complaint of left-sided facial nerve palsy.

Past history

The patient had a history of surgery for left middle ear cholesteatoma performed at another institution: the first one was 38 years ago (at age five), and the second one was 32 years ago (at age 11). Although the surgical details are unavailable, the patient developed profound left sensorineural hearing loss postoperatively. Additionally, the patient was under treatment for schizophrenia.

Present illness

The patient developed left-sided facial nerve paralysis in September of the year preceding the initial consultation (Year X-1), which showed progressive worsening over time. Additionally, the patient developed vertigo. Thus, she was referred to our department in September (Year X) for further evaluation and treatment.

Clinical findings

Upon initial examination, the patient's general status was stable, though she experienced active vertigo. Regarding the facial nerve, she exhibited left-sided facial nerve palsy classified as House-Brackmann Grade III [6], indicating incomplete paralysis. Objective facial nerve assessments, such as electroneurography (ENoG) or electromyography (EMG), could not be performed as the patient refused the examinations due to her underlying schizophrenia. On ear examination, the left external auditory canal was swollen, but the tympanic membrane was intact with no visible perforation. The contralateral right ear showed normal findings. Vestibular assessment revealed non-fatiguable, spontaneous right-beating nystagmus in the non-fixation position. Audiological assessment confirmed profound sensorineural hearing loss in the left ear, while the right ear maintained normal hearing.

Imaging findings

**Figure 1:**
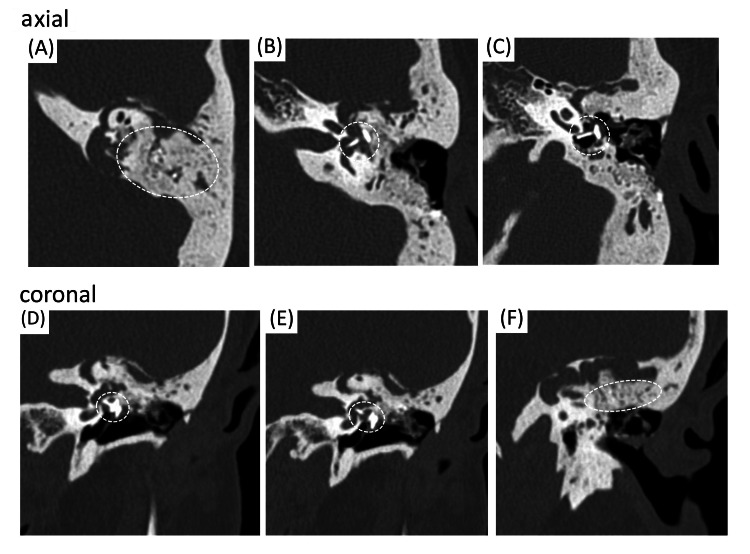
Findings from temporal bone CT A, F: Bone filling material (bone paté), presumably from the previous surgery, extending from the epitympanum to the semicircular canal area. B-E: Ossicular reconstruction material (Apaceram) from the previous procedure was found to be intruding into the vestibule.

Computed tomography (CT) revealed bone filling material (bone paté), presumably from the previous surgery, extending from the epitympanum to the semicircular canal area (Figures 1A, 1F). Ossicular reconstruction material (Apaceram) from the previous procedure was found to be intruding into the vestibule (Figures 1B-1E). Another similar ossicular prosthesis was also identified in the direction of the hypotympanum. Soft tissue density was observed surrounding the rigid filling material in the epitympanum and mastoid antrum. This lesion extended toward the middle cranial fossa, superior to the internal auditory canal, and reached the temporomandibular joint area. Bony dehiscence of the facial nerve canal was also present, suggesting involvement from the geniculate ganglion to the labyrinthine portion of the facial nerve. Magnetic resonance imaging (MRI) with diffusion-weighted imaging (DWI) showed hyperintensity corresponding to the soft tissue density seen on CT, a finding strongly suggestive of cholesteatoma.

Diagnosis and surgical planning

Based on the history, physical examination, and radiological findings, the patient was diagnosed with petrous apex cholesteatoma. The lesion was classified as the supralabyrinthine type according to the Sanna classification [7], extending from the geniculate ganglion of the facial nerve to the superior labyrinthine region and into the deeper petrous apex. Given the progressive facial nerve palsy and the deep extension of the lesion, subtotal petrosectomy was planned for complete excision. Despite the history of hearing loss, the presence of vertigo suggested some residual inner ear function; therefore, we opted for a trans-superior semicircular canal approach in combination with subtotal petrosectomy to reach the medial aspect of the geniculate ganglion while ensuring thorough removal of the lesion.

Surgical findings and procedure

**Figure 2:**
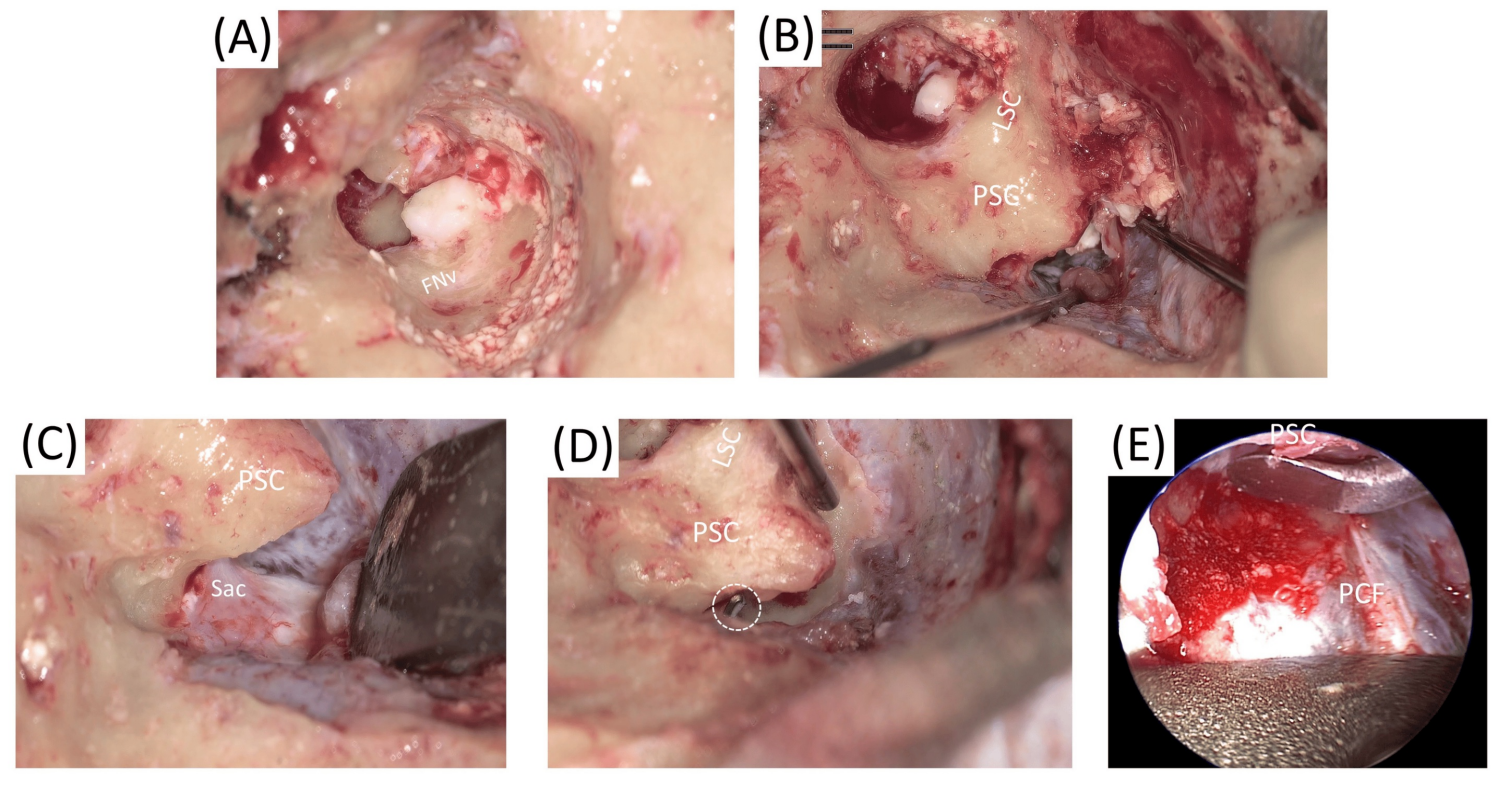
First surgical findings A: The vertical segment of the facial nerve was identified by drilling the posteroinferior wall of the external auditory canal. B: The cholesteatoma matrix was then visualized from the middle cranial fossa side and was sequentially debulked. C, D: The endolymphatic sac was clipped and transected. E: A 30-degree angled endoscope was then inserted between the posterior cranial fossa and the posterior semicircular canal to facilitate the resection of the epithelium in the deep posterior canal region. Abbreviations: FNv: vertical portion of facial nerve, PSC: posterior semicircular canal, LSC: lateral semicircular canal, Sac: endolymphatic sac, PCF: posterior cranial fossa

**Figure 3:**
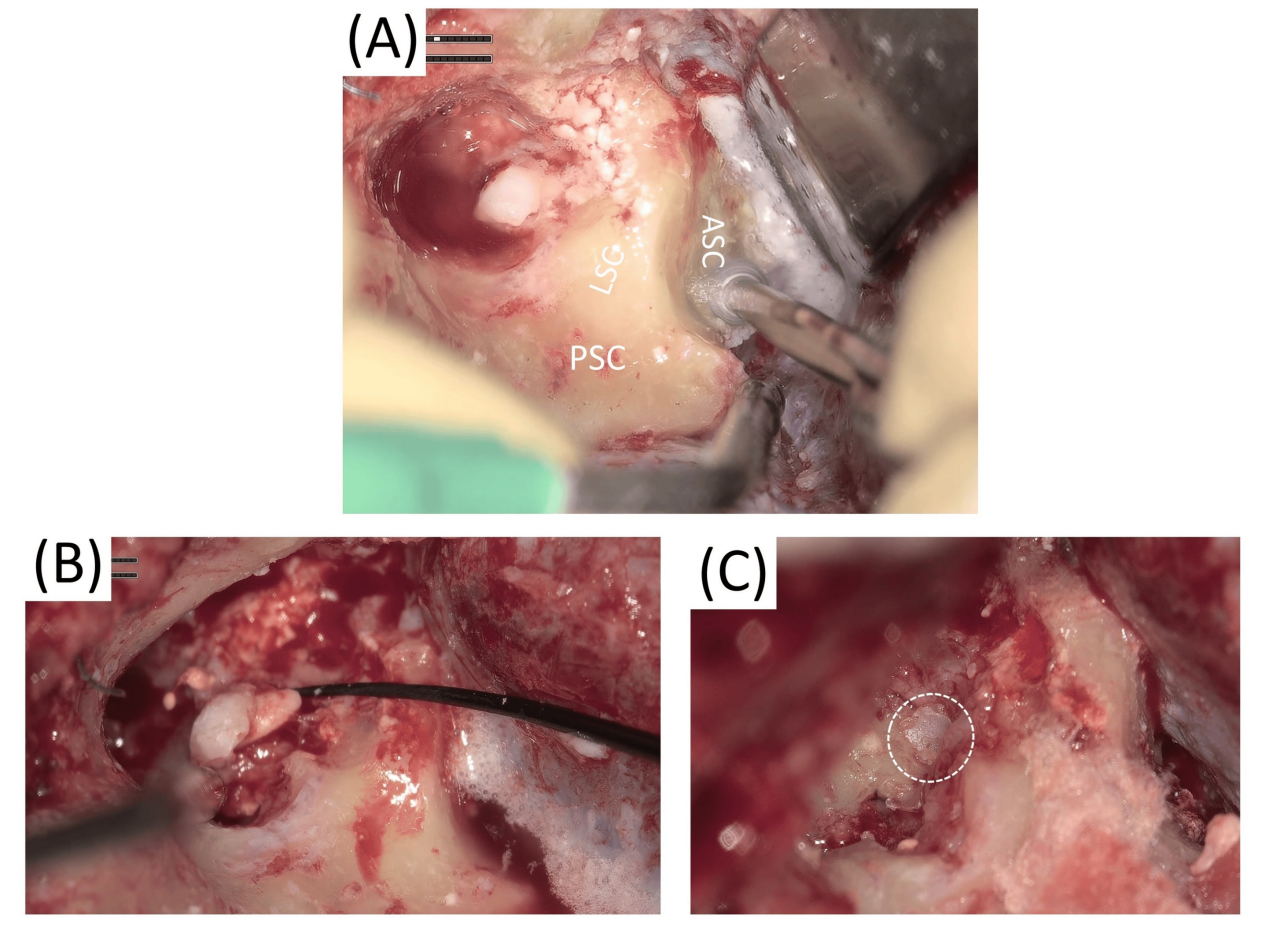
Second surgical findings A: The superior semicircular canal was drilled to expand the surgical access to the deep petrous apex. B-C: The reconstruction material that had intruded into the vestibule through the oval window was carefully extracted. Since the vestibule remained open, it was carefully sealed with bone wax. Abbreviations: PSC: posterior semicircular canal, LSC: lateral semicircular canal, ASC: anterior semicircular canal

**Figure 4:**
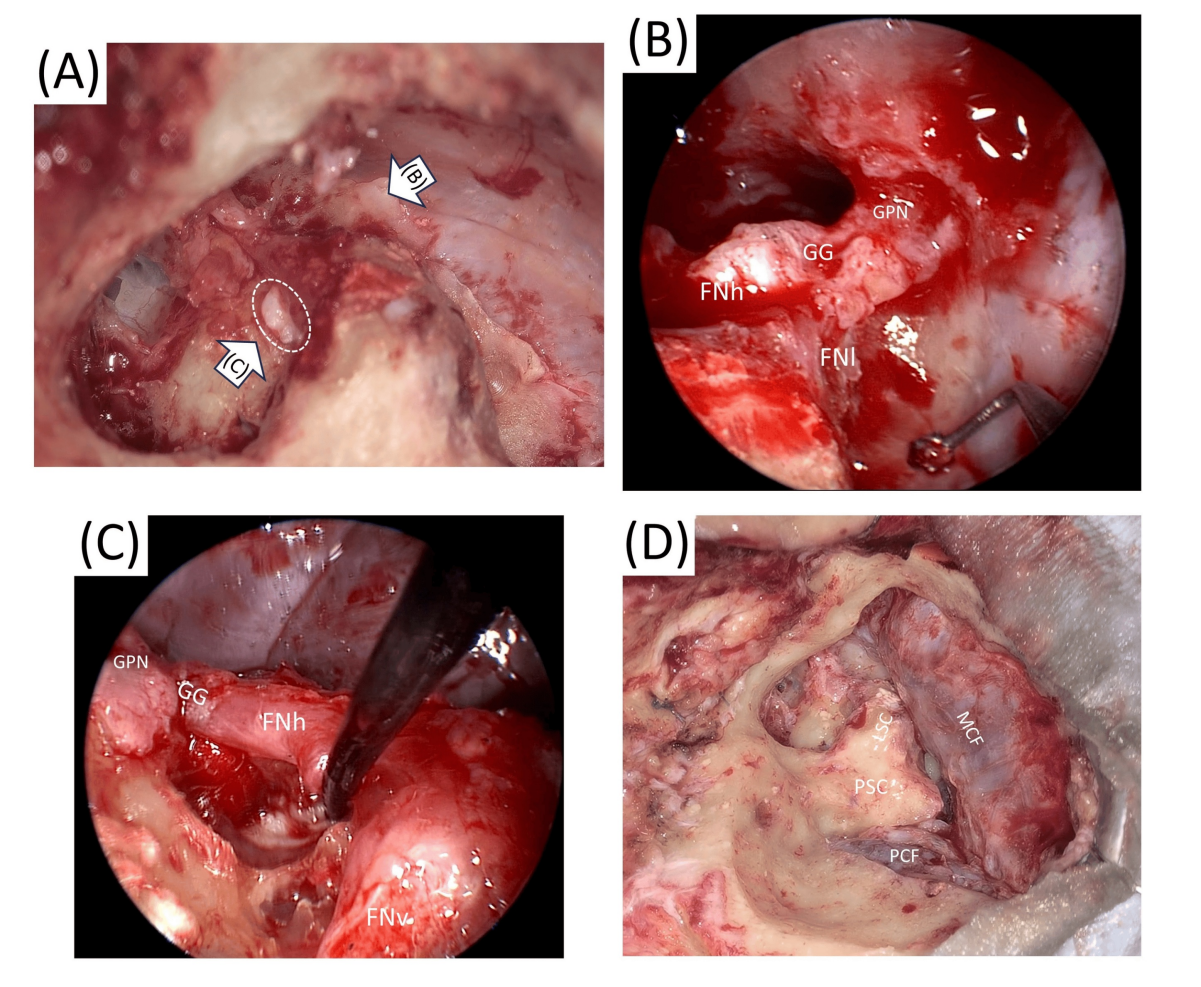
Third surgical findings A, C: When a 30-degree angled endoscope was inserted from the tympanic cavity, the matrix was visualized extending from the medial aspect of the horizontal segment of the facial nerve to the geniculate ganglion, the greater petrosal nerve, and the medial aspect of the labyrinthine portion of facial nerve. B, D: The matrix was completely resected using a combined view from both the tympanic and middle cranial fossa sides. Abbreviations: GPN: greater pyramidal nerve, GG: geniculate ganglion, FNl: labyrinth portion of facial nerve, FNh: horizontal portion of facial nerve, FNv: vertical portion of facial nerve, PSC: posterior semicircular canal, LSC: lateral semicircular canal, Sac: endolymphatic sac, MCF: middle cranial fossa, PCF: posterior cranial fossa

Surgery was performed under general anesthesia in October (Year X). The surgical findings and the procedure are briefly described below (Figures 2-4).

Procedure for the external auditory canal: The external auditory canal was permanently closed using the blind sac closure technique.

Identification of the facial nerve: The skin of the external auditory canal was removed en bloc with the tympanic membrane. Although the tympanic cavity and epitympanum were examined via a transcanal approach, identifying anatomical landmarks was difficult. Consequently, the vertical segment of the facial nerve was identified by drilling the posteroinferior wall of the external auditory canal (Figure 2A).

Mastoidectomy: After exposure of the sigmoid sinus and the middle and posterior cranial fossae, the lateral and posterior semicircular canals were identified using the horizontal segment of the facial nerve as a landmark. The cholesteatoma matrix was identified from the middle cranial fossa side and was sequentially debulked (Figure 2B).

Management of the endolymphatic sac: The epithelium had extended deep to the posterior semicircular canal, with some portions reaching caudal to the endolymphatic sac. The endolymphatic sac was clipped and transected. A 30-degree angled endoscope was then inserted between the posterior cranial fossa and the posterior semicircular canal to facilitate the resection of the epithelium in the deep posterior canal region (Figures 2C-2E).

Trans-superior semicircular canal approach: The superior semicircular canal was drilled to expand the surgical access to the deep petrous apex (Figure 3A).

Management of the oval window: The ossicular reconstruction material lying toward the hypotympanum was removed. The reconstruction material that had intruded into the vestibule through the oval window was carefully extracted. Since the vestibule remained open, it was carefully sealed with bone wax (Figures 3B-3C). Following the removal, the horizontal segment of the facial nerve was clearly identified and confirmed.

Identification and resection of the matrix located medial to the geniculate ganglion: After complete removal of the matrix on the middle cranial fossa side via the trans-superior semicircular canal approach, the geniculate ganglion was exposed. Using a 30-degree angled endoscope inserted from the tympanic cavity, we identified the epithelium extending from the medial aspect of the horizontal segment of the facial nerve to the geniculate ganglion, the greater petrosal nerve, and the medial aspect of the labyrinthine segment (Figures 4A, 4C). The epithelium was completely resected using a combined view from both the tympanic and middle cranial fossa sides (Figures 4B, 4D).

Closure: The eustachian tube was obliterated with bone wax. The mastoid cavity was then packed with abdominal fat, and the surgery was completed.

Postoperative course

Following the surgery, there was a transient, slight worsening of the facial nerve palsy (House-Brackmann Grade III); however, no vertigo or new neurological deficits were observed. At the six-month postoperative follow-up, the facial nerve function had recovered to its preoperative baseline of a House-Brackmann scale of Grade III. No recurrence of the cholesteatoma has been identified to date, as confirmed both clinically and radiologically using MRI.

## Discussion

PAC is a challenging disease due to its deep location. The Sanna classification is widely utilized for surgical planning and risk assessment [7]. Integrating MRI with DWI alongside CT enables precise evaluation of the lesion extent [8]. Our case corresponded to the supralabyrinthine type, which is the most frequent according to Danesi et al. [9].

Selecting a surgical approach requires balancing lesion extent with functional preservation [10]. While lateral approaches suit localized lesions, deep extension often mandates invasive techniques such as the transcochlear approach, where hearing preservation is often not feasible [11,12]. He et al. demonstrated that classification-based strategies achieve high resection rates [13]. We selected the supralabyrinthine approach to access the region surrounding the superior semicircular canal [11,12]. This technique allows access to the petrous apex while preserving the semicircular canal architecture as much as possible, aiding in the maintenance of vestibular function [12]. Furthermore, careful preservation of these structures is essential to minimize the risk of severe inner ear dysfunction, which is another major potential complication in petrous apex surgery.

Management of the facial nerve is critical. Rerouting provides exposure but carries risks. Anterior rerouting has a functional preservation rate of approximately 65-80%, with risks increasing if extensive mobilization is required [3]. Even posterior rerouting increases the risk of transient palsy as the extent of rerouting expands [3]. Llorente et al. emphasized that minimizing neural manipulation is crucial to avoid postoperative dysfunction [3].

However, it must be emphasized that the absolute highest priority in this surgery is the complete eradication of the tumor. While our combined endoscopic and exoscopic approach successfully achieved structural and functional preservation of the facial nerve, surgeons should not hesitate to switch to the conventional facial nerve rerouting approach if excessive tension or burden on the nerve is anticipated during tumor resection. Additionally, in the unfortunate event that the facial nerve is inadvertently severed or must be sacrificed to achieve total tumor clearance, appropriate facial nerve reconstruction (e.g., nerve grafting or primary anastomosis) must be performed to restore function.

Modern device technology has driven surgical advances. Endoscopic ear surgery (EES) contributes to minimally invasive techniques [4]. Angled endoscopes enhance visualization of blind spots, such as the medial aspect of the geniculate ganglion. Marchioni et al. and Shin et al. have highlighted the utility of endoscopy in lateral skull base and petrous tumor surgeries for visualizing hidden regions [14,15]. Furthermore, Iwami et al. reported an "exo- and endoscopic two-step approach" for vestibular schwannoma, utilizing the exoscope for open fields and the endoscope for intracanalicular lesions [16].

EES has advanced minimally invasive temporal bone and skull base procedures [17,18]. The clinical advantages of modern devices and their applications are summarized in Table 1.

**Table 1:** Summary of modern surgical devices and their clinical advantages in skull base surgery

Category	Device/Approach	Key Advantages & Findings	Reference
Endoscopic Surgery	Endoscopic ear surgery/angled endoscopes	Enhances visualization of hidden areas (blind spots) such as the deep petrous apex and the medial aspect of the geniculate ganglion.	Marchioni et al. [14], Sugimoto et al. [18]
Combined Approach	Exo- and endoscopic two-step approach	Combined use for vestibular schwannoma: exoscope for cisternal lesions and endoscope for intracanalicular lesions.	Iwami et al. [16]
				Recent Strategy	"Heads-up surgery" (exoscope + endoscope)	Exoscope secures a wide field while a 30° endoscope visualizes deep structures, minimizes bone removal, and preserves nerves.	Ridge et al. [19]
Current Status	Combined use in the petrous apex cholesteatoma	Reports of combining both devices for this specific pathology remain rare, highlighting the novelty of the present case.	Minoda et al. [5]				
								Present Case	Exoscope + endoscope combined	Achieved complete resection and functional preservation without facial nerve rerouting by overcoming deep blind spots.	This case

Angled endoscopes particularly enhance the visualization of traditional blind spots, such as the petrous apex and the medial geniculate ganglion [14,20]. Beyond cholesteatoma, this utility extends to lateral skull base neoplastic lesions [14,15]. For vestibular schwannoma, the "exo- and endoscopic two-step approach" improves resection precision by using an exoscope for cisternal lesions and an endoscope for intracanalicular lesions [16].

While 3D exoscopes offer high-definition visualization and ergonomic benefits, combining them with endoscopes effectively overcomes deep-seated blind spots. This "heads-up surgery" balances reduced bone removal with nerve preservation [19]. Although reports on this combined use for PAC remain rare [5], our case demonstrates its efficacy. By integrating an exoscope for safe drilling and an endoscope for visualizing the deep medial geniculate ganglion, we achieved complete resection without facial nerve rerouting, successfully balancing neural preservation with tumor removal.

Furthermore, performing the "heads-up surgery" by combining an exoscope and an endoscope in a single stage (one-step procedure) offers significant practical advantages. Compared to conventional standalone microscopic surgery, this combined strategy provides a wider field of view, thereby reducing the risk of residual cholesteatoma. In the present case, a one-step approach was particularly beneficial given the patient's history of schizophrenia, minimizing the overall treatment period and physical burden. By preserving the anatomical structures in their original positions as much as possible, this single-stage surgery minimized postoperative vestibular dysfunction and transient worsening of facial nerve palsy. The lesion in this case was a supralabyrinthine-type PAC, located in an extremely narrow region where the semicircular canals, facial nerve, skull base, and cochlea are densely packed. The rationale for utilizing a 30-degree angled endoscope was that it enabled minimally invasive visualization of these complex, deep-seated structures from both the tympanic and middle cranial fossa sides. Importantly, since both the exoscope and endoscope are utilized in a "heads-up" ergonomic posture, switching between the devices and selecting the optimal endoscope angle can be easily and flexibly tailored to the lesion's specific location and trajectory.

Despite achieving a complete one-step resection, careful and intensive follow-up is critical to assess for recurrence. Since the mastoid cavity was packed with abdominal fat, evaluating residual or recurrent lesions via CT is challenging; therefore, meticulous MRI follow-up is essential. While a standard MRI follow-up protocol after cholesteatoma surgery is generally recommended at one, three, and five years postoperatively [21], we believe that more frequent follow-up is necessary in such complex cases with fat obliteration. If a recurrence is suspected during follow-up, the packed fat can be easily removed. Furthermore, because our minimally invasive approach preserved the anatomical bony landmarks, bone drilling during re-exploration can be performed safely. We believe that performing early re-exploration upon suspicion of recurrence allows for the achievement of a more complete eradication of the disease.

However, it must be acknowledged that this one-step, combined minimally invasive approach requires highly advanced surgical skills and extensive experience with both exoscopic and endoscopic temporal bone anatomy. For general surgeons who do not routinely perform such complex skull base procedures, or in cases where an attempt at complete resection poses an unacceptably high risk of severe complications, adhering to the fundamental principle of exteriorizing the cholesteatoma cavity remains the safest, most practical, and standard alternative.

## Conclusions

We presented a case of supralabyrinthine PAC extending medial to the geniculate ganglion, managed via subtotal petrosectomy combined with a trans-superior semicircular canal approach. In this case, the integrated use of a 3D exoscope for safe bone drilling and a wide operative field, along with a 30-degree angled endoscope, allowed for the clear visualization of lesions in the deep petrous apex and the medial aspect of the geniculate ganglion areas that typically remain blind spots with conventional methods. As a result, we achieved complete resection of the cholesteatoma while avoiding facial nerve rerouting and minimizing the risk of postoperative palsy. The combined use of an exoscope and an endoscope represents an extremely valuable option for balancing neural functional preservation with tumor removal in deep temporal bone and skull base surgery, and its widespread adoption is anticipated in the future.
